# Tooth Transplantation With Sticky Bone for Vertical Bone Defects: A 4‐Year Follow‐Up Case Report

**DOI:** 10.1155/crid/2416769

**Published:** 2026-05-30

**Authors:** Jie Xia, Yuxin Zheng, Runzhi Chen, Fan Yang, Yude Ding

**Affiliations:** ^1^ Department of Stomatology, Center for Plastic and Reconstructive Surgery, Zhejiang Provincial People′s Hospital, Affiliated People′s Hospital of Hangzhou Medical College, Zhejiang, Hangzhou, China; ^2^ School of Stomatology, Zhejiang Chinese Medical University, Hangzhou, Zhejiang, China, zcmu.edu.cn; ^3^ Shangyu Traditional Chinese Medicine Hospital, Shaoxing, Zhejiang, China; ^4^ Department of Stomatology, Hangzhou Geriatric Hospital, Affiliated Hangzhou First People′s Hospital Chengbei Campus, School of Medicine, Westlake University, Hangzhou, Zhejiang, China, westlake.edu.cn

**Keywords:** autologous bone graft, case report, sticky bone, surgical simulation, tooth transplantation

## Abstract

**Background:**

Severe buccal vertical bone defects accompanied by combined periodontal–endodontic lesions are challenging to manage, and evidence regarding the long‐term outcomes of digitally assisted autotransplantation combined with sticky bone grafting remains limited. We aimed to report a case of digitally assisted tooth autotransplantation combined with autologous alveolar bone and sticky bone grafts for the reconstruction of a severe buccal vertical defect.

**Case Description:**

A 23‐year‐old female patient underwent autotransplantation of the right maxillary third molar (#1) to replace the left mandibular first molar (#19) affected by a severe buccal vertical bone defect in July 2020. The autotransplantation procedure involving the donor tooth and the attached autologous alveolar bone from Tooth #1 was simulated using surgical planning software to determine the optimal rotation and angulation of the donor tooth. Buccal bone augmentation for the vertical defect associated with severe furcation involvement was achieved by transplanting the distal alveolar bone of Tooth #1 in combination with sticky bone grafts. The tooth was stabilized with resin fiber tape for 4 weeks.

**Conclusion:**

At the 4‐year follow‐up, no clinical or radiographic abnormalities or signs of infection or inflammation were observed. This approach may represent a feasible option for selected cases with severe buccal vertical bone defects.

## 1. Introduction

Tooth transplantation refers to the transfer of an impacted or erupted tooth from a donor site to an extraction socket or a surgically prepared recipient site, and it is considered a safe and predictable option for replacing missing teeth [1]. In recent years, tooth transplantation has been increasingly applied in clinical practice [2]. The most common indications for tooth transplantation include the replacement of teeth affected by hypoplasia, congenital tooth agenesis, premature or traumatic tooth loss, and severely compromised or nonrestorable teeth [2]. Several studies have suggested that tooth transplantation may offer advantages over alternative treatment options in terms of biocompatibility, function, treatment duration, cost, and long‐term prognosis [3, 4]. Recent systematic reviews have shown that tooth autotransplantation is generally associated with favorable success and survival outcomes, although the overall quality of the available evidence remains limited [5]. Specifically, tooth transplantation can effectively restore dental integrity and masticatory function, making it a highly biocompatible option for replacing missing teeth [3]. Successful transplantation may also promote three‐dimensional (3D) alveolar bone regeneration and gingival papilla formation [6]. Therefore, tooth transplantation is often recommended for growing children and adolescents as an alternative to dental implants, as it preserves and supports continued dentoalveolar growth [6, 7]. Nevertheless, clinical decision‐making becomes more challenging when the recipient site is complicated by chronic inflammation and, in particular, by vertical bone defects, where bone support and periodontal attachment may be compromised.

Clinical studies have indicated that the recipient site for tooth transplantation should provide adequate mesiodistal space, sufficient alveolar bone support, and healthy periodontal attachment, without acute infection or uncontrolled chronic inflammation, to facilitate periodontal tissue regeneration [2, 8]. However, these criteria are largely derived from cases with relatively favorable recipient conditions, and their applicability to recipient sites with compromised periodontal conditions or insufficient bone support remains uncertain [9]. Vertical bone defects—especially buccal vertical defects—often hinder the achievement of adequate bone support and stable periodontal regeneration, which may limit the feasibility of transplantation. Following tooth extraction, implant rehabilitation or other fixed prosthetic restorations are often recommended when recipient sites present with severe furcation involvement combined with chronic apical periodontitis [10]. Generally, tooth transplantation is not considered feasible in severely inflamed or infected recipient sites because of concerns regarding poor prognosis [11]. Nevertheless, recent reports have questioned this traditional view, suggesting that immediate autotransplantation may still be feasible in selected recipient sites with large periapical lesions following thorough debridement and appropriate management, although the currently available evidence remains limited and is based mainly on case reports or small retrospective studies [12, 13]. Therefore, the prognosis of tooth transplantation in the presence of chronic infection, periodontal attachment loss, or vertical bone defects remains controversial. Currently, the efficacy of tooth transplantation in cases of chronic periapical periodontitis, particularly at sites with vertical bone defects, remains unclear. Furthermore, there are no clear clinical treatment guidelines for such cases. Accordingly, additional clinical evidence regarding tooth transplantation in inflamed recipient sites with vertical bone defects, as well as strategies to improve its predictability, is urgently needed.

Therefore, we report a case of a severe buccal vertical bone defect caused by severe periodontal and endodontic lesions, which was treated with tooth transplantation combined with autogenous alveolar bone and sticky bone grafts, aimed at providing a clinical reference for the management of similar cases.

## 2. Case Presentation

**Figure 1:**
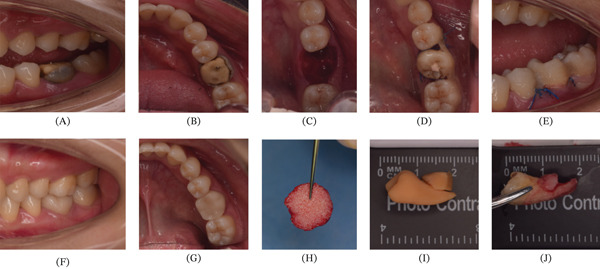
Autologous alveolar bone and sticky bone grafting during maxillary third molar autotransplantation. (A, B) Preoperation intraoral view of the recipient site (#19), (C) recipient site after preparation, (D, E) immediate placement of the donor tooth (#1) stabilized by resin fiber tape, (F, G) buccal and occlusal view at 48 months, (H) sticky bone (i − PRF + bovine bone substitute), (I) 3D tooth‐bone replica, and (J) donor Tooth #1 with distal alveolar bone.

**Figure 2:**
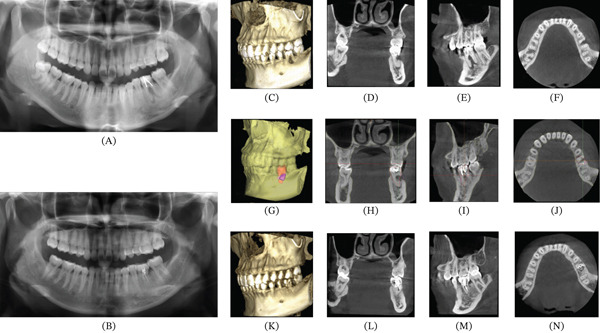
Panoramic radiograph, CBCT and virtual planning of tooth autotransplantation. (A) Preoperative panoramic radiograph; (B) Postoperative panoramic radiograph; (C) Preoperative 3D reconstruction: severe buccal bone loss at #19; (D–F) preoperative CBCT coronal/sagittal/axial views: buccal vertical bone loss and pulpal floor perforation and chronic apical periodontitis at Tooth #19; (G) virtual planning: donor Tooth #1 with bone graft; (H–J) Virtual autotransplantation on CBCT coronal, sagittal, and axial views; the red: Tooth #1, the pink: bone graft; (K) 3D reconstruction at 48 months; (L–N) 48‐month CBCT coronal/sagittal/axial views: buccal bone regeneration and intact lamina dura and continuous PDL space.

A 23‐year‐old female patient presented to the outpatient clinic complaining of chewing discomfort accompanied by a recurrent abscess in the left mandibular posterior region for more than 3 years. The patient reported having received prior dental treatment for pain 10 years earlier, but the exact details were unknown. Oral examination revealed Tooth #19 as a residual crown with Grade I percussion pain and Grade I mobility. Pulp sensibility tests (cold test and electric pulp testing) were performed, and Tooth #19 showed no response. Deep buccolingual periodontal pockets (> 4 mm) were present, with positive bleeding on probing (BOP) and a gingival index (GI) score of 2, and horizontal probing extended through the furcation area. Furcation involvement was classified as Grade III. Several restorations were noted on the occlusal surface (Figure 1). Tooth #1 had vertical eruption, whereas Tooth #32 had distoangular impaction covered by a distal gingival flap. A panoramic radiograph (Figure 2) and cone‐beam computed tomography (CBCT) (Figure 2) showed that Tooth #19 had no history of root canal treatment and probable apical pathology as well as buccal vertical bone resorption. Imaging showed that Tooth #19 had been previously repaired with postretained restorations. Furthermore, CBCT images suggested perforation of the pulpal floor (Figure 2).

The final diagnosis of the presented case was a residual crown of Tooth #19 with Grade III furcation involvement, suspected perforation of the pulpal floor, chronic apical periodontitis, and impacted Tooths #1 and #32.

## 3. Treatment

### 3.1. Preoperative Assessment

After a detailed evaluation of the case, the following treatment options were recommended to the patient: (1) root canal treatment, perforation repair, surgical periodontal therapy, and buccal vertical bone regeneration of Tooth #19 and (2) extraction of Tooth #19 followed by implant restoration or autotransplantation of Tooth #1 to the site of Tooth #19. The advantages and disadvantages of each option were analyzed, and the patient was informed about the potential risks and complications. Considering the possible complications, treatment costs, and long‐term prognosis, the patient opted for tooth transplantation. The transplantation surgery was scheduled for July 2020. Root canal therapy for Tooth #1 was initiated 1 week before surgery.

### 3.2. Surgical Procedure

After disinfection and local anesthesia, Tooth #19 was extracted, and the inflammatory granulation tissue in the extraction socket was curetted. Moreover, a severe vertical bone defect was observed on the buccal side of the recipient site. Subsequently, the rongeur was used to remove the bone block after the final preparation of the recipient site was verified using the 3D tooth and bone replica. A blood sample was drawn from the forearm and collected into two noncoated orange cap tubes of 10 mL each. The blood in the tubes was used to prepare injectable platelet‐rich fibrin (i‐PRF) by centrifugation at 2700 rpm (650 × g) for 3 min. Next, the i‐PRF was mixed with bovine bone substitute (Geistlich Bio‐Oss, Geistlich Pharma, Wolhusen, Switzerland) to form a stabilized fibrin‐based bone graft composite, often termed “sticky bone.” A collagen membrane (Geistlich Bio‐Gide, Geistlich Pharma, Wolhusen, Switzerland) was placed between the buccal mucosal flap and the buccal bone plate. The space in the buccal defect was filled with the prepared sticky bone. To minimize periodontal ligament (PDL) damage, Tooth #1 was extracted using a minimally invasive approach, accompanied by a part of the distal alveolar bone. The donor was rotated to ensure that the attached distal alveolar bone was located on the buccal side of the recipient site. After the transplant was pressed into place, its buccal position, root insertion depth, and contact with neighboring teeth were checked to ensure that the transplant was fully in the correct position. Furthermore, the transplant was adjusted until no occlusal contact with the opposing teeth was observed. Eventually, the gingival flap was tightly sutured around the transplant. The tooth was kept in position with resin fiber tape for 4 weeks (Figure 1).

### 3.3. Postoperative Care and Follow‐up

Suture removal was carried out 2 weeks postoperatively. Four weeks later, splint removal, periodontal probing, marginal gingival assessment, and tooth mobility assessment were performed. Additionally, the transplanted tooth was monitored with clinical and radiographic follow‐up at 6 months and 1 year. At follow‐up, the transplanted tooth showed favorable periodontal conditions, with probing depths of less than 3 mm, healthy marginal gingiva, and no abnormal mobility. The radiograph revealed that the transplant had good apical and periradicular bone healing 6 months after the operation. The crown restoration was completed 6 months after transplantation. Radiography and CBCT revealed obvious vertical bone regeneration at the buccal aspect of Tooth #19. Meanwhile, a normal PDL space with no pathological signs of root resorption was also observed. A concise timeline of the case presentation, treatment, and follow‐up is summarized in Table 1.

**Table 1:** Timeline of case presentation, treatment, and follow‐up.

Time point	Key events
10 years before presentation	Root canal therapy for pain.
> 3 years before presentation	Recurrent abscesses and chewing discomfort in the left mandibular posterior region.
Initial visit	Tooth #19: residual crown, Grade III furcation involvement, suspected pulpal floor perforation, chronic apical periodontitis, and buccal vertical bone loss; Tooth #1 selected as the donor tooth; Tooth #32 with distoangular impaction.
1 week before surgery	Root canal therapy for donor Tooth #1.
July 2020 (surgery)	Extraction and debridement of Tooth #19; recipient site preparation with 3D replica guidance; i‐PRF preparation and sticky bone grafting; autotransplantation of Tooth #1 with distal alveolar bone; collagen membrane placement; resin fiber tape splinting.
2 weeks postoperatively	Suture removal.
4 weeks postoperatively	Splint removal; periodontal and mobility assessment.
6 months postoperatively	Radiographic follow‐up; crown rehabilitation.
1 year postoperatively	Clinical and radiographic follow‐up.
48 months postoperatively	Clinical examination and CBCT: asymptomatic; no percussion pain or mobility; intact lamina dura and periodontal ligament space; buccal bone augmentation; no infection or inflammation.

## 4. Outcome and Follow‐Up

At the 48‐month follow‐up, the patient was asymptomatic. Clinical examination revealed that the transplanted tooth was stable, without percussion pain or abnormal mobility. Periodontal evaluation demonstrated probing depths of < 3 mm, healthy marginal gingiva without bleeding on probing, and no pathological mobility. Healthy gingival soft tissue was maintained throughout the follow‐up period (Figure 1). The CBCT examination confirmed the absence of infectious or inflammatory signs. The lamina dura and an intact PDL space were observed on CBCT images (Figure 2). CBCT also demonstrated favorable outcomes of tooth transplantation and vertical bone augmentation on the buccal side of the recipient site at 48 months after surgery (Figure 2).

## 5. Discussion

Tooth transplantation is a well‐established option for tooth replacement [1, 2, 4]. Reported success rates range from 79% to 100% [6]. Moreover, compared with dental implants, a transplanted tooth can preserve proprioception and the PDL and allow continued physiologic dentoalveolar development [14]. However, when the recipient site is chronically inflamed, particularly in the presence of severe buccal bone defects, bone augmentation is often required prior to implant placement [15]. Consequently, the prolonged treatment timeline associated with two‐stage implantation may deter patients from choosing this approach. Therefore, in the present case, tooth transplantation was considered an appropriate treatment option. In clinical practice, third molars can be transplanted to first or second molar sites when the original teeth are difficult to preserve due to caries or trauma [2]. Immediate transplantation can be performed after tooth extraction in sites with severe furcation involvement and chronic apical periodontitis, which may substantially reduce overall treatment time [12]. Moreover, concomitant autogenous bone grafting can be performed when the donor is a maxillary third molar, as the distal maxillary tuberosity region provides abundant bone. Animal studies have shown that PDL stem cells can contribute to the formation of PDL‐like structures in periodontal defects [16, 17]. New alveolar bone formation may occur when PDL stem cells are preserved on the root surface, which may be critical for successful transplantation [16]. Transplanting the maxillary third molar with distal alveolar bone can preserve more PDL stem cells and facilitate the process of autogenous bone grafting. Consequently, a maxillary third molar with attached distal alveolar bone was selected as the donor in this case. By mobilizing the distal bone plate of the maxillary tuberosity during extraction of the maxillary third molar, a donor tooth with attached autologous alveolar bone was obtained, which may provide additional PDL stem cells.

Digital technologies, such as 3D reconstruction and surgical simulation, may improve the efficiency and precision of transplantation by reducing surgical time [18]. Moreover, surgical simulation can help determine an optimal 3D position for the transplanted tooth, thereby reducing trial‐fitting time and minimizing potential iatrogenic mechanical injury to PDL cells [18]. Therefore, we performed preoperative surgical simulation for transplantation of the maxillary third molar together with alveolar bone from the maxillary tuberosity region and fabricated a 3D tooth‐bone replica, which helped identify bony interference during transplantation. By using the 3D replica, we extracted the affected tooth and removed the interference, which reduced the extraoral time of the donor third molar and may have contributed to the favorable outcome of transplantation.

Regarding reconstruction of the buccal vertical defect, autologous bone grafting has been reported, although graft resorption remains a concern [19]. In case reports, combining autologous bone grafting with tooth transplantation has been described as a potential approach for managing vertical bone defects at nonrestorable molar sites [20, 21]. However, although vertical bone volume increased after autologous bone block grafting compared with the preoperative baseline, vertical bone resorption was still observed at 6 months postoperatively in these reports. Autologous bone grafts have several drawbacks, including technique sensitivity, graft‐related complications, and unpredictable graft resorption [22]. Postoperative bone volume resorption is a common and well‐recognized issue after autologous bone grafting [23]. In the field of bone augmentation, Aboelela et al. introduced the sticky bone technique in 2015 as a fibrin‐based bone graft composite entrapped within a fibrin network [24]. Moreover, sticky bone can be readily molded into the desired shape, facilitating handling and helping prevent graft dispersion [20]. One study suggested that filling the defect with sticky bone and covering it with an autologous PRF membrane may accelerate bone formation and wound healing [25]. Hence, the combination of autologous bone and sticky bone may represent a useful strategy to reduce postoperative bone resorption in the present case.

In the present case, long‐term follow‐up supported the stability of this combined approach. This case suggests 4‐year stability of a combined approach using an autologous tooth–alveolar bone graft and a sticky bone composite for reconstruction of a severe buccal vertical defect, for which long‐term data remain limited. By comparing preoperative and 4‐year postoperative coronal CBCT images, significant vertical bone augmentation was observed on the buccal side of the autotransplanted tooth. In line with the concept of “dedicated” dental CBCT, CBCT acquisition should be tailored to the diagnostic task and optimized to minimize radiation exposure (e.g., a small FOV and appropriate low‐dose protocols) [26].

However, as this is a single case and standardized quantitative outcomes (e.g., probing depth, mobility grading, and CBCT‐based bone measurements) were not consistently available, further studies with larger cohorts and more standardized outcome assessments are warranted to validate these findings.

## 6. Conclusion

In conclusion, the maxillary third molar with distal alveolar bone combined with sticky bone grafts in tooth transplantation may be a feasible option for the treatment of teeth with severe periodontal and endodontic lesions accompanied by buccal vertical bone defects. Before surgery, the application of digital technologies, including 3D virtual simulation and surgical simulation, may help minimize the extraoral time and the fitting attempts required to insert the donor tooth into the recipient socket. Sticky bone with an autogenous bone graft can provide an effective treatment for vertical bone augmentation in the recipient site. Therefore, it may provide a less invasive treatment option for tooth transplantation in the buccal vertical bone defect area. Nevertheless, future studies with long‐term follow‐up and larger sample sizes are necessary to explore the potential advantages of this technique.

## Author Contributions

Jie Xia: conceptualization, methodology, and writing—original draft. Yuxin Zheng: conceptualization, methodology, and writing—original draft. Runzhi Chen: methodology and software. Fan Yang: investigation and writing—review and editing. Yude Ding: conceptualization, validation, methodology, and writing—review and editing. Jie Xia, Yuxin Zheng, and Runzhi Chen contributed equally to this work and share first authorship.

## Funding

No funding was received for this manuscript.

## Disclosure

All authors have read and approved the final manuscript.

## Ethics Statement

This case report has been reviewed by the Ethical Committee of the Ethical Committee permitted the case report.

## Consent

Written informed consent was obtained from the patient for future publication of the case report and any accompanying images.

## Conflicts of Interest

The authors declare no conflicts of interest.

## Data Availability

The data that support the findings of this study are available from the corresponding author upon reasonable request.
